# Hypergraphia in temporal lobe epilepsy

**DOI:** 10.4103/0972-2327.56323

**Published:** 2009

**Authors:** Giridhar P. Kalamangalam

**Affiliations:** Department of Neurology, University of Texas Health Science Center, Houston, TX 77030, USA

**Figure 1:**
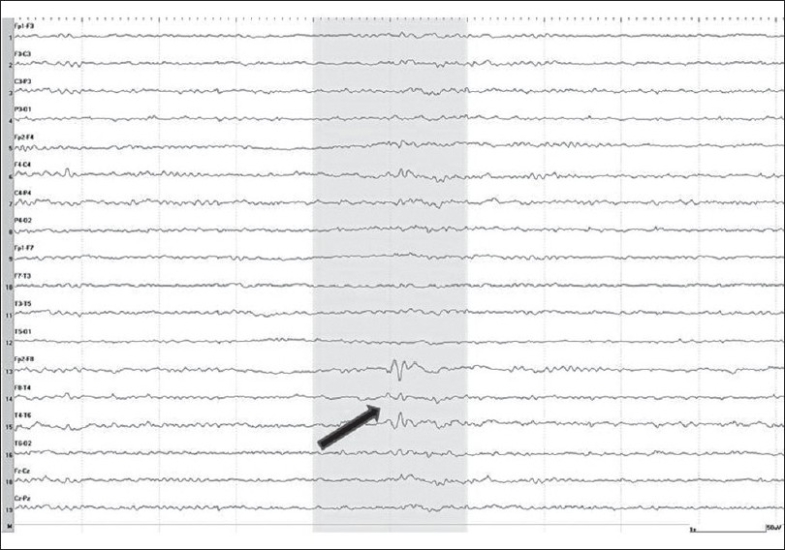
Sleep EEG, bipolar longitudinal montage: a single spike maximum over the right anterior temporal region (arrow)

**Figure 2:**
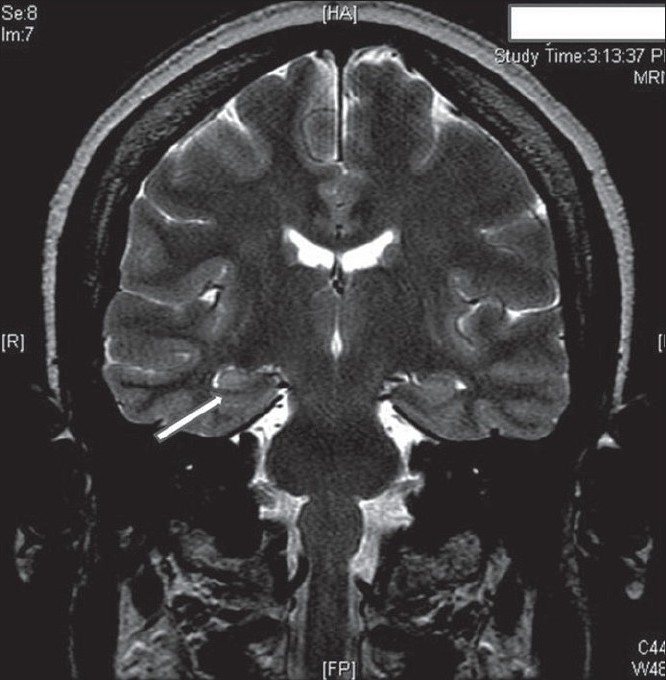
1.5T MRI: T2-weighted coronal view, showing right hippocampal sclerosis (arrow)

A 44-year-old right-handed female presented with a history suggesting temporal lobe epilepsy since the age of 11. She was admitted to the monitoring unit for characterization of seizures. Interictal electroencephalography (EEG) showed frequent right temporal spikes in sleep [Figure 1]. Multiple auras of a rising abdominal feeling were recorded, many with right temporal EEG change. Two complex partial seizures with proximal and distal manual automatisms were also recorded, with bitemporal EEG change. Magnetic resonance imaging (MRI) of brain showed right hippocampal sclerosis [Figure 2].

**Figure 3:**
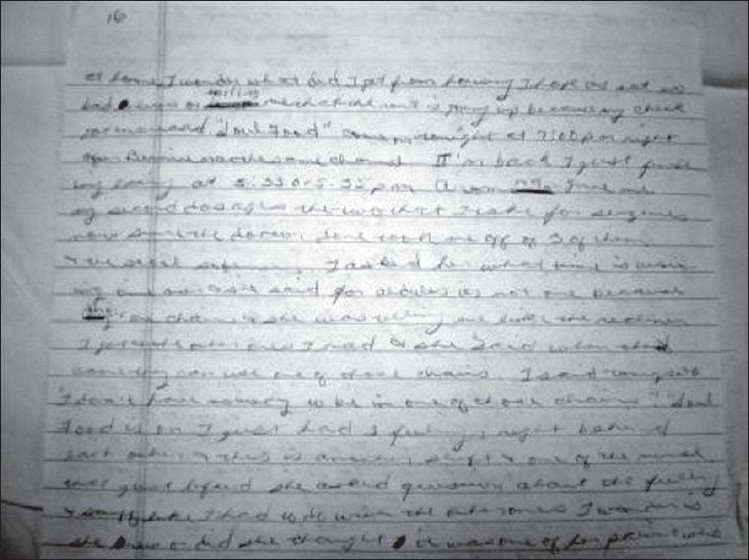
Close up of page 16. The patient writes about her medication dosing, including minor details

The patient was observed to write for much of her waking hours. The document, a letter to her husband, numbered 29 pages by the time of her discharge from hospital and remained unfinished. The writing was cramped, dense, and used all the available space on both sides of the sheet, including the margins. The contents of the letter were rambling, though with specific details. She wrote about her hospital stay, often mentioning exact times, her intake of medications, and minor details of conversations with staff. In the interests of the patient's privacy, a more detailed examination was thought inappropriate, though she permitted limited photography of her writing [Figure 3]. In later conversation, the patient related that she always ‘liked writing’, either in longhand or on a typewriter, and had considered a career as an office secretary. Her friends teased her that she habitually carried around paper and a clipboard.

In a now classic article, Waxman and Geschwind[1] described a tendency to excessive, and sometimes compulsive, writing in patients with temporal lobe epilepsy. The eponymous ‘Geschwind syndrome’ denotes this, together with previously-described reports of hyperreligiosity, sexual dysfunction and tendency to pedantry (‘stickiness’) in such patients. Though the syndrome as a neuropsychiatric entity subsequently attracted controversy,[2] many neurologists will recognize facets of related behavior in their patients with temporal lobe epilepsy.
